# Histopathology of Growth Anomaly Affecting the Coral, *Montipora capitata*: Implications on Biological Functions and Population Viability

**DOI:** 10.1371/journal.pone.0028854

**Published:** 2011-12-19

**Authors:** John H. R. Burns, Misaki Takabayashi

**Affiliations:** Marine Science Department, Tropical Conservation Biology and Environmental Science, University of Hawai'i at Hilo, Hilo, Hawai'i, United States of America; Institute of Marine Research, Norway

## Abstract

Growth anomalies (GAs) affect the coral, *Montipora capitata*, at Wai'ōpae, southeast Hawai'i Island. Our histopathological analysis of this disease revealed that the GA tissue undergoes changes which compromise anatomical machinery for biological functions such as defense, feeding, digestion, and reproduction. GA tissue exhibited significant reductions in density of ova (66.1–93.7%), symbiotic dinoflagellates (38.8–67.5%), mesenterial filaments (11.2–29.0%), and nematocytes (28.8–46.0%). Hyperplasia of the basal body wall but no abnormal levels of necrosis and algal or fungal invasion was found in GA tissue. Skeletal density along the basal body wall was significantly reduced in GAs compared to healthy or unaffected sections. The reductions in density of the above histological features in GA tissue were collated with disease severity data to quantify the impact of this disease at the colony and population level. Resulting calculations showed this disease reduces the fecundity of *M. capitata* colonies at Wai'ōpae by 0.7–49.6%, depending on GA severity, and the overall population fecundity by 2.41±0.29%. In sum, GA in this *M. capitata* population reduces the coral's critical biological functions and increases susceptibility to erosion, clearly defining itself as a disease and an ecological threat.

## Introduction

Reports of coral diseases have increased over the last several decades along with concerns that changing environmental conditions are promoting disease susceptibility [1]–[3]. A substantial effort has been made to describe coral diseases around the globe, however comprehensive pathological characterizations, ecological drivers, and the associated threat to particular populations or communities is undetermined in many cases [3]–[6]. As environmental stressors in coastal areas are predicted to intensify, increasing disease prevalence may exacerbate synergistic threats to coral reef ecosystems [7], [3], [8]. It is therefore critical to characterize coral diseases comprehensively to determine their impacts on organismal function, population viability, and coral reef ecosystem integrity.

Growth anomaly (GA) is a coral disease first recorded in 1965 that has now been identified in multiple species from reefs throughout the world [9]–[11]. GAs have been characterized by reduced or absent polyp formation, depletion of symbionts, hyperplasia of the basal body wall, reduced growth rates, and decreased skeletal density [12], [13], [10]. GAs are generally easily identifiable as irregular protuberant masses that differ dramatically in morphology from nearby unaffected tissue [14]–[17]. However, gross morphology and cellular characteristics of GAs can exhibit intra- and inter-specific variation [18], [12], [10]. The variability in GA characteristics highlights the need for an in-depth study of this disease in each affected coral species. Etiology, transmissibility, pathogenesis, and mortality associated with this disease in most coral species remain largely uncharacterized [13], [19], [10], [17].

Systematically describing lesions in conjunction with histopathology is critical for coral disease research as it enables development of case definitions and identification of causal agents [15], [20], [21], [22]. Histological analyses of healthy, stressed, or diseased coral tissue can provide clues on potential impacts to the host such as feeding, immune and physical defense mechanisms, growth, and reproduction [15], [23], [24]. Given that one of the key signs of GA is anomalous skeletal growth, this disease's impacts on coral skeletal density and crystallography also need to be investigated to determine the susceptibility of the affected coral colony to erosion and predation [25], [26].

**Figure 1:**
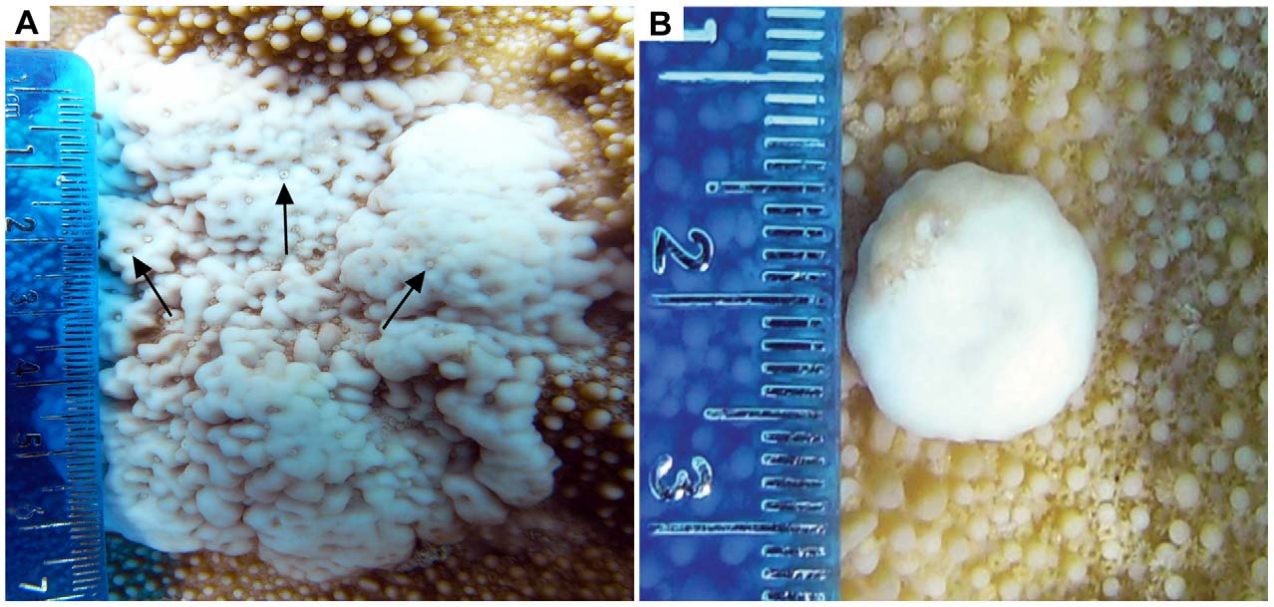
Type A and Type B GA morphology. Photographs of Type A and Type B GA tissue **a.** Type A GA, note reduction in polyps (arrows) and fused protrusive tuberculae. **b.** Type B GA, note lack of polyps and fused protuberant coenosteum.

The Rice Coral, *Montipora capitata*, inhabiting the Wai'ōpae tide pools, southeast Hawai'i Island, display an abnormally high level of GA prevalence compared to other surveyed sites throughout the Hawaiian archipelago [27]–[30], [17]. Two distinguishable forms of GA affect *M. capitata* at this site, Type A and Type B (Figure 1), with prevalence of 22.1% and 8.2%, respectively [17]. Type A GA show significant reduction in polyp and tubercular density with many tuberculae being fused and protruded while Type B GAs have no discernable polyps and fused and protuberant coenosteum [17]. Evidence from prior epizootiological analysis supports the hypothesis of pathogenesis from Type A to Type B morphology [17]. To better understand GA in *M. capitata*, we examined their cellular and skeletal pathology. Specifically, our aims were to: 1) histopathologically analyze the cellular characteristics of Type A and B GAs compared to healthy and unaffected (apparently healthy tissue from a diseased colony) *M. capitata* tissue; 2) analyze the density and crystalline structure of skeleton deposited by healthy and affected tissue; and 3) develop a quantitative assessment of the impact of GA at the colony and population level by collating histopathological data with previously collected epizootiological data on the severity and prevalence of GAs in the same *M. capitata* population.

## Methods

### Sample Collection

All samples for this study were collected at Wai'ōpae (19°29′55″N 154°49′06″W), southeast Hawai'i Island, in tide pools that fall outside of the Wai'ōpae Marine Life Conservation District (MLCD). Coral fragments (<2 cm diameter) were collected from *M. capitata* colonies in collaboration with the State of Hawai'i Department of Land and Natural Resources, Division of Aquatic Resources. Coral tissues were sampled 2 days prior to a projected spawning event in order to ensure the presence of ova in tissues. Prior to sampling, reference photographs were taken of the *M. capitata* colonies using a Sea & Sea DXIG underwater camera (Tabata Inc., Long Beach CA, USA) with a wide-angle (16 mm) conversion lens. Tissue biopsy samples were collected by scuba using stainless steel cores. Samples were taken from 6 Type A and 6 Type B GA lesions from the central portion of 12 *M. capitata* colonies. For each GA-affected colony, unaffected tissue (apparently healthy tissue on a diseased colony) was sampled adjacent (proximal) to the GA and at the periphery (distal) of the colony for a total of 24 unaffected samples. Additional healthy tissue samples were taken in duplicates from central and peripheral parts of 3 colonies devoid of GAs. All tissue samples were preserved in Zinc-Formaldehyde solution (Z-Fix, Anatech Ltd., Battle Creek MI, USA) diluted 1∶4 in filtered seawater [10], [21].

Small skeletal fragments were removed from each sample for X-ray diffraction analysis. Skeletal fragments from an additional 12 samples (3 healthy, 3 unaffected, 3 Type A, 3 Type B), taken for separate symbiotic dinoflagellate genotyping and density analyses, were used for computerized tomography (CT) analysis. The coral tissue in samples used for skeletal analyses was removed by soaking in sodium hypochlorite solution (10%) overnight, and the resulting coral skeleton was gently but thoroughly rinsed in deionized water.

### Histopathology

All preserved coral tissue samples were photographed, rinsed, and decalcified with Formical-2000 (Decal Chemical Corporation, Tallman NY, USA). Decalcified samples were trimmed, placed in standard processing cassettes, and stored in 70% ethanol. Samples were dehydrated and processed using a LEICA TP-1020 (Leica Microsystems Inc., Buffalo Grove IL, USA) tissue processor by sequential submersions in 70% ethanol for 60 min, 95% ethanol for 60 min, 100% ethanol for 60 min, followed by clearing in CitriSolv (Thermo Fisher Scientific Inc., Waltham MA, USA) for 120 min. Samples were infiltrated with molten paraffin wax at 70°C for 120 min and poured into standard molds. Serial sagittal sections were cut at 4 µm thickness using a rotary microtome, placed on clean glass slides and stained with hematoxylin and eosin. Slides were examined using light microscopy at magnifications ranging from 4–100×.

Cellular and tissue characteristics were quantified to compare healthy, proximal and distal unaffected, Type A GA, and Type B GA coral tissues. Ova, sections of mesenterial filaments, nematocytes, and symbiotic dinoflagellates within the gastrodermis were counted along contour lengths of coenenchyme [10]. Due to sampling 2 days prior to a projected spawning event, ova were mature and clearly distinguishable from developing spermaries. Counts of each histological feature were normalized to contour length [10] and are referred to as density values for each feature (ovum density, mesenterial filament density, nematocyte density, and symbiont density). Evidence of hyperplasia of the basal body wall, necrosis, and invasion of filamentous fungi or algae that were reported in previous histological examination of GAs [12], [20], [10] were also examined.

### X-Ray Diffractometry

X-ray diffraction (XRD) analysis was carried out at room temperature using a Bruker D8 AXS system (Bruker GmbH, Karlsruhe, Germany). All experiments were conducted using a monochromated Cu Kα radiation point source (λ = 1.5406 Å) at an operating voltage and amperage set, respectively, to 40.0 kV and 40.0 mA. The coral skeleton samples were loaded in a 0.1 mm low-absorption borosilicate glass capillary and rotated throughout the data collection period. Homogenized skeleton from healthy, unaffected (distal & proximal), Type A, and Type B GA samples were scanned at a scanning range between 20 and 60 degrees with a scanning rate of 0.5/min, and a step size of 0.02 for quantification. Diffraction patterns were compared between samples to known mineral compositions using JADE XRD analysis software (Materials Data Inc., Livermore CA, USA).

### Computerized Tomography

Each coral skeleton sample denuded of tissue as described above was completely submerged in deionized water in a plastic cup and tapped lightly to remove air bubbles from all the pores to avoid density artifacts [31]. The density of coral skeleton relative to water was obtained with a computed tomography (CT) scan (Aquilion 32, Toshiba, Hawai'i Radiologic Associates 146 Ltd.) at scan thickness of 0.5 mm, 120 kV and 250 mA, rotation time of 0.5 S, and results expressed as Hounsfield units. The mean density in Hounsfield Units was measured for each of ten 0.5 mm-thick transverse scans along a 5 mm distance from the basal body wall using Vision Reach analysis software (AMICAS Inc., Boston MA, USA).

### Calculations for Effects of GA on Biological Functions at Colony and Population Levels

The impact of GA on biological functions at colony and population levels was determined by collating histological data with the previously collected epizootiological data from the same coral population at Wai'ōpae [17]. First, reductions in densities of ova, symbiotic dinoflagellates, mesenterial filaments and nematocytes in Type A and Type B GA compared to healthy tissues were determined from the histological analyses. Impacts at the colony level were determined by multiplying GA-induced reductions in densities of histological features by the GA severity (proportion of colony surface area occupied by GA) of affected colonies. The mean Type A and Type B GA severity among all surveyed colonies, previously collected from an exhaustive survey of 1093 *M. capitata* colonies in the same population [17], was used to estimate the reduction in densities of ova, symbiotic dinoflagellates, mesenterial filaments and nematocytes at the population level.

### Statistical Analysis

Density data derived from the histological and skeletal analyses were transformed, if necessary using log and arcsine transformations, to meet the assumptions of normality and equal variance required for use of parametric statistical tests. Variation in mean values of cellular and skeletal characteristics was analyzed among the examined tissue types using a one-way multivariate analysis of variance (MANOVA). The data were further evaluated using univariate ANOVA and Tukey's HSD post hoc tests to determine statistical differences (α = 0.05) among the examined tissue types. All statistical tests were run using Minitab 15 (Minitab Inc., State College PA, USA) statistical software.

## Results

### Histopathology

**Figure 2 pone-0028854-g002:**
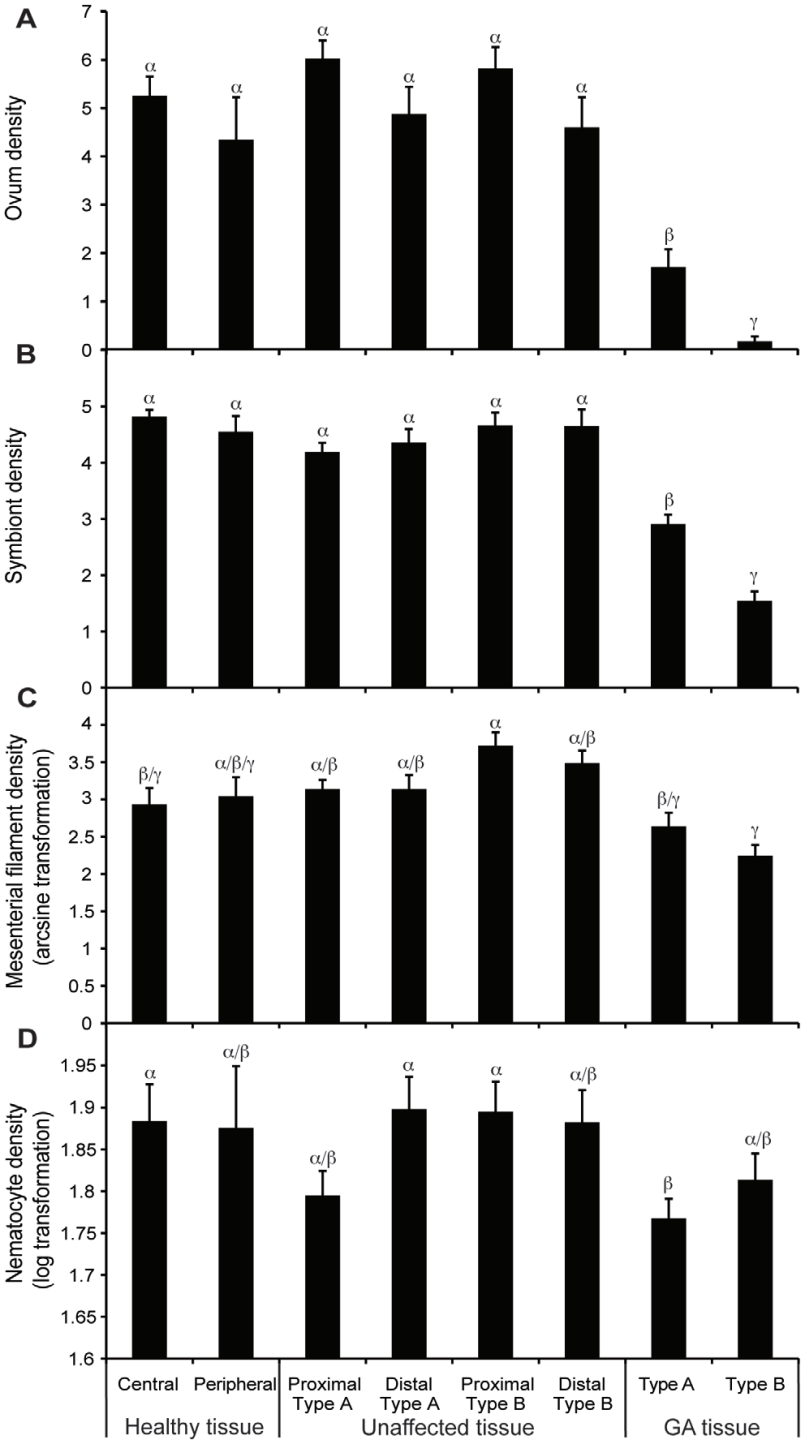
Histological comparison of *M. capitata* tissue conditions. Histological comparison among healthy central (n = 3), healthy peripheral (n = 3), unaffected proximal to Type A (n = 6), unaffected distal to Type A (n = 6), unaffected proximal to Type B (n = 6), unaffected distal to Type B (n = 6), Type A GA (n = 6) and Type B GA (n = 6) tissues of the coral, *Montipora capitata*. **a.** ovum density (mean ± S.E.); **b.** symbiotic dinoflagellate density (mean ± S.E.); **c.** mesenterial filament density (mean ± S.E.); and **d.** nematocyte density (mean ± S.E.). α, β, and γ denote groupings identified by statistical significance (p<0.01).

Densities in histological features were significantly different among the analyzed tissue types (MANOVA, F = 12.50, p<0.001; Table 1). Further univariate analyses showed that density of ova and symbionts were significantly lower than healthy and unaffected tissues for Type A and Type B GAs. Type B GA had significantly lower ovum and symbiont densities than Type A (ANOVA, F = 28.96–34.67, p<0.001; Tukey's HSD p = 0.05; Figure 2a,b). Density of mesenterial filaments was lower in Type A and Type B GAs (Figure 2c) than healthy and unaffected tissues. Only Type B GA had significantly lower density of mesenterial filaments compared to healthy and unaffected tissues (ANOVA, F = 6.41, p<0.001; Tukey's HSD p = 0.05). Nematocyte density was lower in Type A GA, Type B GA, and unaffected tissue proximal to Type A lesions (Figure 2d). Only Type A GA tissue had significantly lower nematocyte density compared to healthy central tissue, unaffected tissue distal to Type A, and unaffected tissue proximal to Type B lesions (ANOVA, F = 2.38, p<0.001; Tukey's HSD p = 0.05).

**Table 1:** Statistical results from MANOVA and ANOVA analyses.

MANOVA				
Source	Wilk's lamba	F-ratio	df	P
Tissue type	0.211	12.50	28	0.000

ANOVA				
Dependent variable		F-ratio	df	P
Ovum density		28.96	7	0.000
Symbiont density		34.67	7	0.000
Mesenterial filament density		6.41	7	0.000
Nematocyte density		2.38	7	0.024

Results from MANOVA and ANOVA analyses of the examined tissue conditions. Relevant test statistics are provided along with resulting p-values. All p-values are significant (<0.05).

**Figure 3 pone-0028854-g003:**
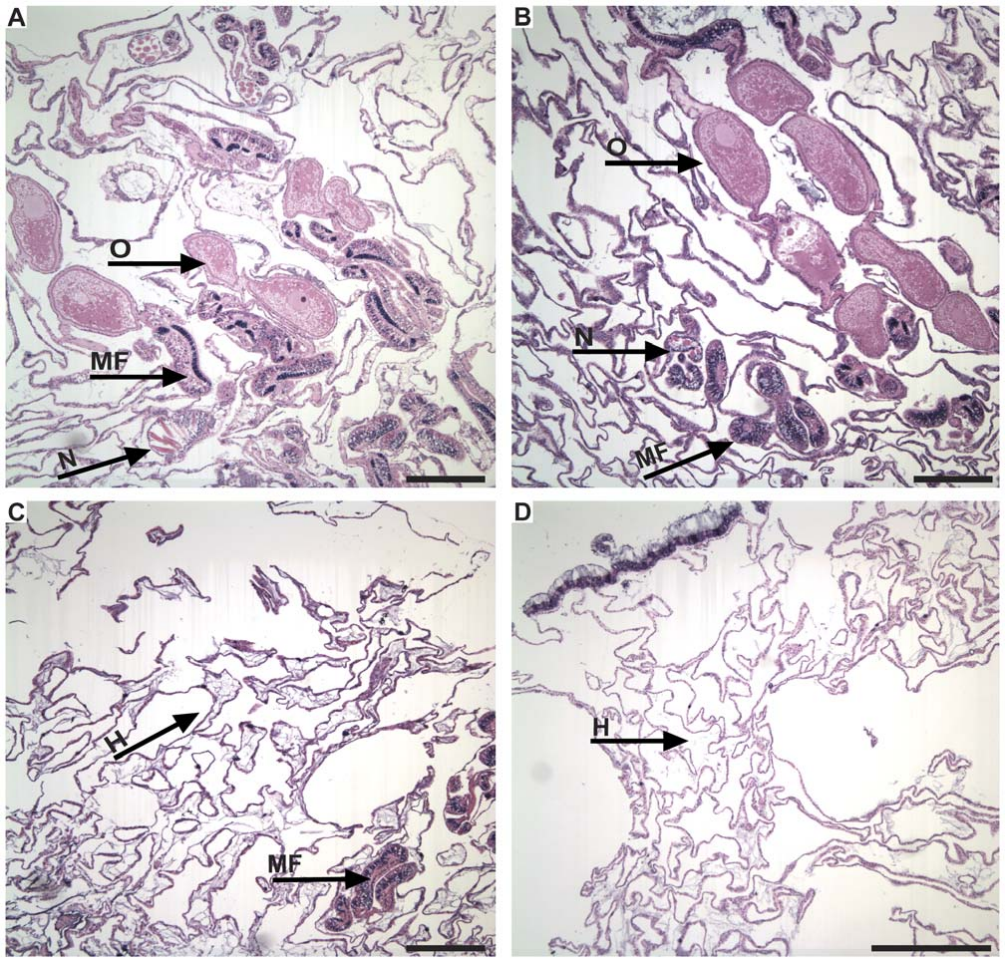
Sagittal histological sections. Sagittal histological sections of the coral, *Montipora capitata*. **a.** Healthy tissue. Note the developing ova (O), mesenterial filaments (MF), and nematocytes (N). **b.** Unaffected tissue directly adjacent to GA. Note the developing ova (O), mesenterial filaments (MF), and nematocytes (N). No evidence of hyperplasia or cellular irregularity is present. **c.** Type A growth anomaly (GA). Note the hyperplasia and disorganization of the basal body wall (H), reduced numbers of mesenterial filaments (MF), and absence of ova. **d.** Type B GA. Note complete absence of ova and mesenterial filaments with marked hyperplasia (H) of the basal body wall. Bars = 250 µm.

Sagittal sections of Type A GA tissue showed hyperplasia of the basal body wall with a corresponding reduction in mesenterial filaments and nematocytes as well as symbiotic dinoflagellates (Figure 3c). Type B GA tissues consistently exhibited severe hyperplasia of the basal body wall (Figure 3d). Mesenterial filaments and nematocytes as well as symbiotic dinoflagellates were present in Type B GA tissue yet located deeper in the aboral regions of the tissue than healthy and unaffected samples. Neither Type A nor B GA tissue showed atypical amounts of necrosis nor algal/fungal invasion compared to healthy and unaffected coral tissue.

### X-Ray Diffractometry

All homogenized skeletal samples from each tissue condition showed identical diffraction patterns. The patterns matched that of pure aragonite and showed no evidence of other minerals or changes to the crystalline structure.

### Computerized Tomography

**Figure 4 pone-0028854-g004:**
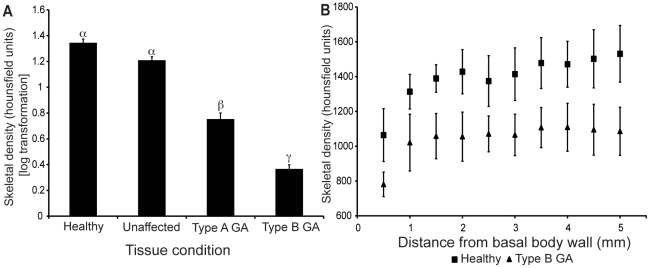
Comparisons of skeletal density. Comparison of skeletal density, measured by CT scan, of *Montipora capitata*. **a.** Mean (± S.E. n = 12) densities of the 5 mm skeletal layer bordering the basal body wall. **b.** Serial densities through 0.5 mm-thick transverse sections from the basal body wall in Healthy and Type B growth anomaly (GA). α, β, and γ denote groupings identified by statistical significance (p<0.01).

GA had significantly lower skeleton density than healthy and unaffected tissues with Type B having significantly lower density than Type A (ANOVA, F = 149.59, p<0.001, Tukey's HSD p = 0.05, Figure 4a). Both Type A and B GA skeletal densities were consistently lower in all serial transverse sections compared to healthy samples. However, only Type B skeletal densities were significantly lower in all serial transverse sections compared to healthy samples (ANOVA, F = 10.86–25.89, p<0.001, Tukey's HSD p = 0.05, Figure 4b).

### Impacts to Biological Functions and Viability at Colony and Population Levels

**Figure 5:**
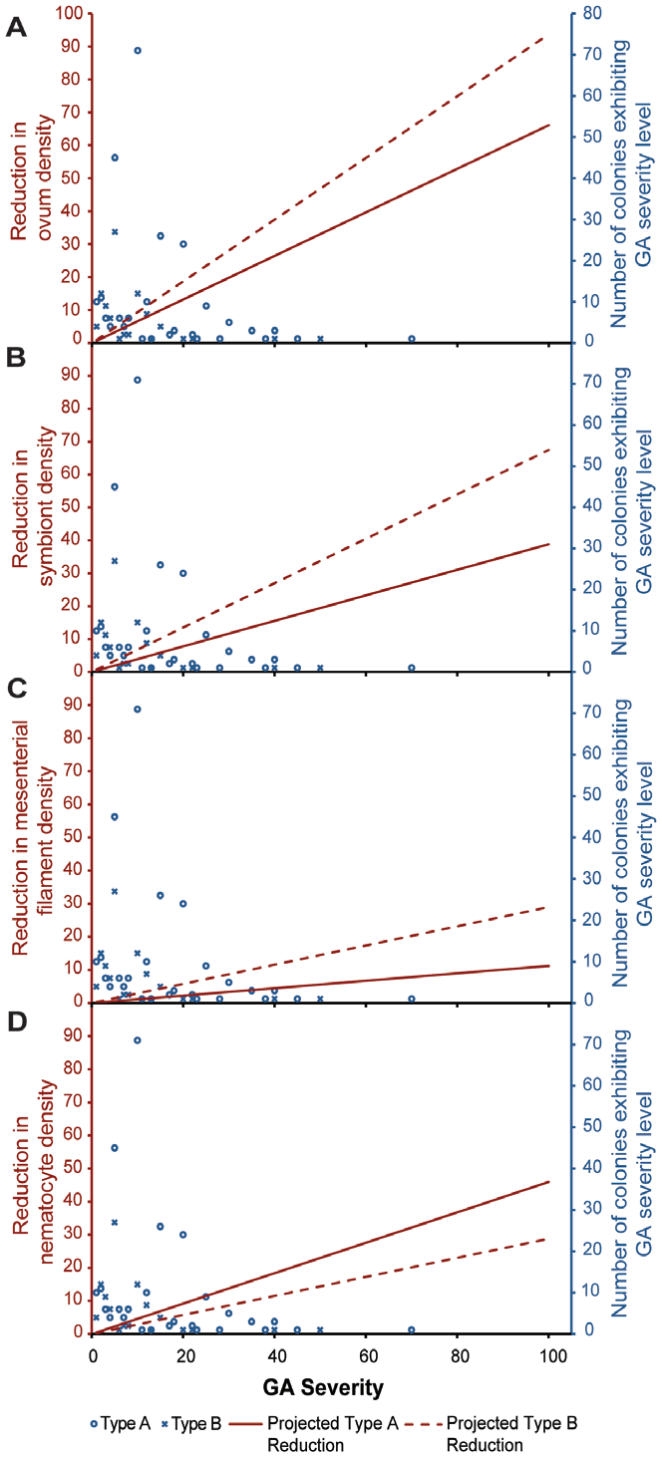
Projections of GA impacts at the colony level. Colony-level reductions (left Y-axis) projected by a function of GA severity (proportion of colony surface area occupied by GA, x-axis) and the absolute frequencies of GA severities in the population (right Y-axis). Projections are shown for both Type A and Type B GAs for **a.** ova, **b.** *Symbiodinium*, **c.** mesenterial filaments, and **d.** nematocytes.

The above histological analyses showed that the densities of ova, symbiotic dinoflagellates, mesenterial filaments, and nematocytes were reduced by 11.2–93.7% in GA tissues. The reductions in densities of these four histological features can be projected at the colony level by modeling the calculated reductions against GA severity (Figure 5). Further collating the above histological data with previously collected GA severity data from the 1093 surveyed colonies (relative cover of 2.80±0.2% for Type A and 0.60±0.09% for Type B) [17], the population-wide reductions in the densities of ova, symbiotic dinoflagellates, mesenterial filaments, and nematocytes were estimated to be 2.41±0.29%, 1.49±0.17%, 0.49±0.29%, and, 1.46±0.18% respectively (Table 2).

**Table 2:** Impacts of GA on *M. capitata* tissue characteristics at the population level.

	Reduction in ovum density	Reduction in symbiotic dinoflagellate density	Reduction in mesenterial filament density	Reduction in nematocyte density	Population severity (mean±S.E.)
*Histological Analysis*					
Type A	66.1%	38.8%	11.2%	46.0%	2.8±0.2%
Type B	93.7%	67.5%	29.0%	28.8%	0.60±0.09%
*Estimated impacts to population based on GA severity data* (mean±S.E.)	2.41±0.29%	1.49±0.17%	0.49±0.29%	1.46±0.18%	

Summary of the reductions in densities of ova, symbiotic dinoflagellates, mesenterial filaments, and nematocytes seen in Type A and B growth anomalies (GAs), compared to the healthy tissue from histological analysis. Mean values of Type A and Type B severity are shown for all colonies surveyed in this population (n = 1093). The reductions in these parameters were combined with GA severity data (right column, from Burns et al. 2011) to estimate their impacts at the population level (bottom row).

## Discussion

Coral GA lesions are easily identified on the basis of gross morphology as they develop into pale protuberant masses, but the etiology, pathogenesis, and physiological impacts of this disease are poorly understood [6], [32], [17]. This study histopathologically characterized Type A and Type B growth anomaly (GA) in the tissue and skeleton of the coral, *Montipora capitata*, from Wai'ōpae, southeast Hawai'i Island (Figure 1). *M. capitata* corals at this site exhibit an unusually high prevalence level of this disease [17], [28]. Impacts of GA to the cellular and skeletal characteristics of affected coral colonies were examined in order to quantitatively assess the threat of this disease.

The GA lesions in *M. capitata* occur more frequently in the central portion of coral colonies than the periphery [17]. The central and peripheral tissues of healthy colonies were compared to determine if histological differences between GA and non-GA tissue might be due to spatial location within a colony. Histology of healthy *M. capitata* colonies was determined to be spatially homogenous (Figure 2), thus verifying that any anomalous histological characteristics found in GA lesions were due to the disease. Departures from healthy conditions in GA lesions were seen in gross morphology and densities of several cellular features. All findings from this investigation support the growing evidence that GA significantly affects coral's biological function and is, by definition, a disease [33]. Furthermore, Type B GA tissue showed the most prominent disease signs as elucidated below (Figures 1–5), marking it as the more severe and advanced stage of this disease than Type A, therefore supporting our hypothesis of pathogenesis from healthy to Type A GA then to Type B GA [17].

The histological features of *M. capitata* GAs observed in this study have been previously characterized for coral GAs affecting other species. Hyperplasia of the basal body wall, which seems to be an intrinsic characteristic of GA tissue in all corals previously studied [12], [20], [13], [10], was commonly seen in the examined *M. capitata* GAs. Interestingly, such hyperplasia was more distinct and apparent in Type B than Type A (Figure 3c–d). In contrast to other studies, we saw no consistent evidence of necrosis or fungal/algal invasion in GA tissues of *M. capitata* [34], [12], [5], [10].

Several lines of histopathological evidence clearly demonstrated that GA lesions affect the cellular composition of the coral, *M. capitata*. First, densities of nematocytes (cells producing nematocysts that deliver toxins to either entangle prey or repel attackers) and mesenterial filaments (cells involved in digestion and protection) were reduced in both Type A and B GAs (Figure 2), compared to healthy or unaffected tissues. These findings are consistent with other investigations of GAs affecting other coral species and indicate that biological functions involved in feeding, digestion, defense, and prey capture are compromised in both Type A and B GAs [12], [13], [10]. Intriguingly, the densities of mesenterial filaments and nematocytes did not differ between Types A and B GAs. However, functionality of these tissues are likely to be more impaired in Type B since polyp tentacles and perisomatic openings to the gastrovascular cavity are completely absent in this type of GA in *M. capitata* [17].

Symbiotic dinoflagellates translocate photosynthetically derived energy to the coral hosts, and thus density of symbiotic dinoflagellates is an indicator of the amount of energy made available for coral [35]. The density of symbiotic dinoflagellates was significantly reduced in GA tissue, especially in Type B, compared to healthy and unaffected tissues (Figure 2). Such loss of algal symbionts appears to be a common characteristic of GA tissue [12], [36], [20], [13], [10]. Metabolic capacity and growth rates of coral housing GAs may therefore be severely impaired [34], [37], [38]. The lack of symbionts within the oral region of GA tissue may also explain the translucency of these lesions [10], giving them a “bleached” appearance.

Ovum development is essential for reproductive capability and is directly linked to reproductive fitness of an organism. The significantly reduced ovum density in GAs compared to healthy and unaffected tissue (Figure 2) elucidates the detrimental impact of this disease on reproductive potential. Further, ovum density was significantly lower in Type B GA than Type A, the former having almost no ova among all tissue sections examined (Figure 2). Previous studies have also found reduced ovum development in GA lesions affecting other coral species [12], [13]. Given that ovum production represents the majority of energetic demand of spawning coral reproduction, this finding clearly indicates this disease not only affects the fecundity of individual *M. capitata* colonies, but also cause reductions of the overall reproductive potential of an affected coral population as discussed later.

Since the most prominent morphological feature of GAs is the protrusion of coral skeleton, the crystallography of skeleton was analyzed to determine if GA impacts the integrity and composition of the CaCO_3_ deposited by the calicoblastic epithelial cells. X-ray diffraction patterns of the skeletal samples from each tissue condition were consistent with that of pure aragonite skeleton. Knowing disease tissue produces aragonite skeleton, we can reject the possibility that altered CaCO_3_ crystalline structure causes the amorphous appearance of the GA lesions. This finding also supports previous investigations showing GA lesions do not impact skeletal CaCO_3_ crystalline structure [9], [14]. Contrastingly, skeletal density was significantly reduced in GAs compared to healthy and unaffected samples (Figure 4a). Reduced skeletal density in GA lesions will likely increase susceptibility of affected colonies to erosion and predation especially because the density reduction is maintained in skeleton bordering the basal body wall (Figure 4b).

All histological parameters in tissues unaffected by GAs from colonies that housed GAs elsewhere resembled those of healthy colonies, regardless of whether the unaffected tissues were proximal or distal to GA lesions. Therefore we can conclude that the histological abnormalities observed in GA tissue were confined to the visible lesions (Figure 2). Since the histological anomalies of GA are limited to lesions, we can estimate the impact of GA at the colony and population level of this coral population using previously collected epizootiological data (Table 2, Figure 5). Although the mean severity of GA (expressed as proportion of colony surface area occupied by GA) in this population was relatively low (2.8±0.2% for Type A and 0.60±0.09% for Type B) [17], the functional impairment at the population level, indicated by reduced densities of ova, symbiotic dinoflagellates, mesenterial filaments, and nematocytes, was appreciable. For example, GA induces a reduction in fecundity at the population level by 2.41±0.29% (Table 2). Additionally, it is important to note that some individual colonies had as much as 70% surface area occupied by Type A GA (Figure 5). Individuals succumbing to such high levels of relative GA cover can lose nearly half of their reproductive potential. This may be detrimental to the fitness of genotypes carried by these individual colonies in evolution of this coral population.

Quantifying impacts of any coral disease at the colony and population level is a crucial part of future research in the face of global increase in prevalence (number of cases of a disease in a given population at a given time) and severity (proportion of colony surface area occupied by coral disease) of many coral afflictions. Collecting severity data is necessary for making estimates of disease impacts at a larger scale. Collating physiological analyses with severity data for any health affliction can enable quantification of impacts across broader scales. These types of assessments are not achievable with only disease prevalence data. Including additional biological and ecological parameters that are affected by diseases in the analyses will further improve the capability of estimating the overall impacts of any coral disease in the future.

While this study showed that GA impacts were confined to the lesions in *M. capitata*, the threat of this disease should not be dismissed. Longitudinal photo monitoring over 3 years in this population suggest that the typical prognosis of GA-affected *M. capitata* is no recovery, and there is very limited evidence of tissue regeneration [28]. The disease pathogenesis from Type A to Type B, that is now well supported, suggests that the compromised functionality due to this disease should only be worsening with time. Investigation of incidence rate and recovery will provide better estimations of the mortality risk posed to the examined coral population. This study highlights the importance of combining comprehensive epizootiological data with histological analyses in order to assess the threat of a coral disease. Future studies should continue to investigate etiology in conjunction with environmental stressors associated with GA prevalence so that causal factors of this disease can be identified.
